# Functional specificity of rat vibrissal primary afferents

**DOI:** 10.14814/phy2.12810

**Published:** 2016-06-10

**Authors:** Facundo A. Lucianna, Fernando D. Farfán, Gabriel A. Pizá, Ana L. Albarracín, Carmelo J. Felice

**Affiliations:** ^1^ Laboratorio de Medios e Interfases (LAMEIN)Universidad Nacional de TucumánSan Miguel de TucumánArgentina; ^2^ Departamento de Bioingeniería, Facultad de Ciencias Exactas y TecnologíaInstituto Superior de Investigaciones Biológicas (INSIBIO)CONICET – UNTSan Miguel de TucumánArgentina

**Keywords:** Peripheral afferent activity, ROC analysis, specificity, vibrissae

## Abstract

In this study, we propose to analyze the peripheral vibrissal system specificity through its neuronal responses. Receiver operating characteristics (ROC) curve analyses were used, which required the implementation of a binary classifier (artificial neural network) trained to identify the applied stimulus. The training phase consisted of the observation of a predetermined amount of vibrissal sweeps on two surfaces of different texture and similar roughness. Our results suggest that the specificity of the peripheral vibrissal system easily permits the discrimination between perceived stimuli, quantified through neuronal responses, and that it can be evaluated through an ROC curve analysis. We found that such specificity makes a linear binary classifier capable of detecting differences between stimuli with five sweeps at most.

## Introduction

Understanding how neurons represent, process, and manipulate information at the peripheral level is of crucial interest, because it allows speculating capabilities and limitations of the sensory system (Victor 2006). Thus, the first problem that the neuroscientist must face is to determine if a specific stimulus set applied to a biological system produces specific, precise and well‐differentiated responses (Arabzadeh et al. 2006; Adibi and Arabzadeh 2011; Pizá et al. 2014).

Discriminability measures have been derived from the information theory (Shannon 1948) and the signal‐detection theory (Green and Swets 1989), and these have allowed quantifying the response reliability (Chichilnisky and Rieke 2005). Specificity and sensitivity are measures that can be used to evaluate the decoding capability of the sensory system. Specificity is an indicator of the ability of the sensory system to differentiate distinct stimuli. On the other hand, sensitivity is the ability of the system to respond correctly to variations in the stimulus of the same type. This study aims to establish a functional characterization of the vibrissal system through the analysis of specificity.

Rodent vibrissae are specialized hairs that, due to the presence of sensitive mechanoreceptors at their follicles, provide accurate somatosensory input (Bosman et al. 2011). When vibrissae move along a texture, their trajectories are characterized by irregular, skipping motions (slip‐stick events: high velocity jumps over texture grains) (Wolfe et al. 2008; Jadhav and Feldman 2010). Follicular receptors transform the mechanical events (slip‐sticks) into electrical activity, spikes, that are then transmitted through multiple axons (infraorbital nerve) to higher levels (Bezdudnaya and Castro‐Alamancos 2011; Bosman et al. 2011). Previous studies have shown that it is possible to identify specific discharge patterns in vibrissal primary afferents, which would allow discrimination of rough surfaces (Albarracín et al. 2006; Arabzadeh et al. 2006; Farfán et al. 2011, 2013). However, there is not a quantification of functional characteristics, such as the specificity of the system.

According to the signal‐detection theory, a system with high level of specificity and sensitivity would be able to classify and/or identify the source of neuronal responses after observing a few responses (Green and Swets 1989). In this study, we propose to measure the neuronal responses specificity using the receiver operating characteristics (ROC) technique (Fawcett 2006). We found that the specificity of the peripheral vibrissal system easily permits the discrimination between perceived stimuli and that it can be quantified through an ROC curve analysis (area under an ROC curve). It has also been found that such specificity makes a linear binary classifier capable of detecting differences between stimuli with five sweeps at most.

## Materials and methods

### Experimental data

Experimental data were obtained by Albarracín et al. (2006). Briefly, five Wistar adult rats (300–350 g) were used. Also, bipolar electrodes, placed on the Gamma vibrissal nerve, were used to record the afferent activity during the sweeping of Gamma vibrissa on different textures. The vibrissa movement was induced by electrical stimulation of the facial motor nerve. A slip‐resistance level was presented by placing the surface at a maximal distance from the whisker base so that the tip just barely contacted the surface throughout the entire movement cycle.

**Figure 1:**
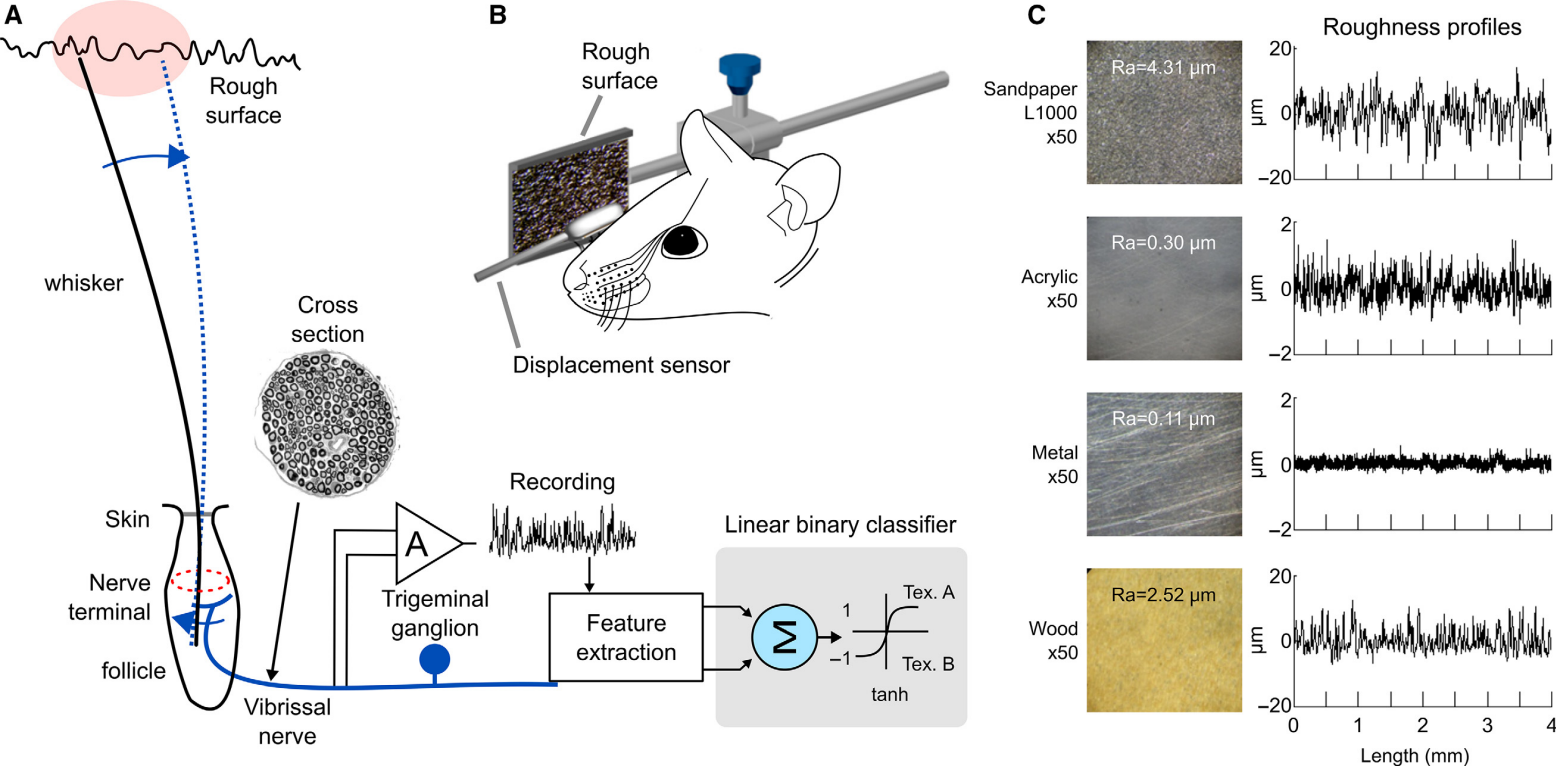
Procedure for functional specificity measure, experimental set up and stimuli set. (A) Schematic diagram of the peripheral transduction mechanism. Contact between whisker and surface, during active sweep, evokes kinetic signatures characterized by slip‐stick events which are encoded by spikes trains that are conduced by vibrissal nerve fibers. The multifiber activity is recorded and amplitude and spectral features are extracted. Then, a linear binary classifier identifies the stimuli origin (after learning process) (B) Spatial disposition of rough surfaces and vibrissal movement monitoring. (C) Photographs of sweep surfaces and roughness profile measurements obtained using a Hommel Tester T1000 (Hommel Werke). The Ra parameter (arithmetical deviation of the assessed profile) is a roughness estimation (International Standards BS.1134 and ISO 468).

The swept surfaces were wood, metal, acrylic, and sandpaper L1000 (Fig. 1C). Three surfaces (wood, metal and acrylic) were polished using the same grade sandpaper L1000 to obtain similar roughness and different textures. Surface texture is not a measurable quantity; it is not possible to assign a unique “texture” value to every different surface. However, it is possible to measure some of the intrinsic characteristics, or parameters, of surface texture. Thus, the surfaces roughness was measured using a Hommel Tester T1000 (Hommel Werke, www.hommel-etamic.de). Roughness profiles and Ra parameter (a specific roughness feature of International Standards BS.1134 and ISO 468) are shown in Figure 1C.

Only data between 5 and 100 ms were taken into account. This procedure does not include the discharges related to the muscular activation, and only the data obtained when the vibrissa was sweeping surfaces were processed. We consider all sweeps recorded for our analysis. The root mean square (RMS) value was used as estimator parameter of the signal energy (Challis and Kitney 1990). The Power Spectrum Density (PSD) was calculated using the Burg parametric estimation method (Pardey et al. 1996). Both methods were applied to all recordings, obtaining 50 RMS and 50 PSD values for each sweep surface. The PSD were represented using the maximum frequency (fmax) which is defined as the maximum‐energy frequency component. It was calculated within the range of 100 to 600 Hz. Thus, a dataset is obtained of 200 cases and 3 variables: the two measures and the label of the surface material. The theoretical details of both processing techniques can be found in Albarracín et al. (2006) and Farfán et al. (2011).

All these procedures were done in accordance with the recommendations of the Guide for the Care and Use of Laboratory Animals (National Research Council, NRC).

### Specificity quantification

For each combination of surface pairs, we generated several binary classification models using a simple perceptron (SP) as classification algorithm (Hertz et al. 1991). SPs were trained using the same amount of features for each surface. For each SP, we randomly selected 25 cases per surface to test the prediction capacity of the model. Of all the remaining features 1, 2, 3, 4, 5, 10, 15, 20, and even 25 cases were randomly taken for training. This process was repeated 100 times. Thus, 900 SPs were generated for each one of the six surface combinations.

As supervised training we used the gradient descendent algorithm (Hertz et al. 1991). The inputs were the RMS and fmax values scaled between −1 and 1 using the minimum and maximum values of cases corresponding to the pair of surfaces to be classified. We used the hyperbolic tangent function as sigmoid output function.

Furthermore, we used ROC curves (Receiver Operating Characteristic) (Fawcett 2006) to assess the predictive power of SPs generated using the outputs of the classifications made on features chosen to the test them. We used the statistical AUC (area under the ROC curve) as a summary measure of the ROC curve; this statistic is a measure of the overall performance of classification and it is independent of the threshold value used to perform a binary classification (Fawcett 2006). We implemented the algorithms 1 and 2 of Fawcett (2006) for generating the ROC curves and calculating AUC, respectively. For the algorithmic implementation, we used GNU Octave 3.6.4.

## Results

**Figure 2:**
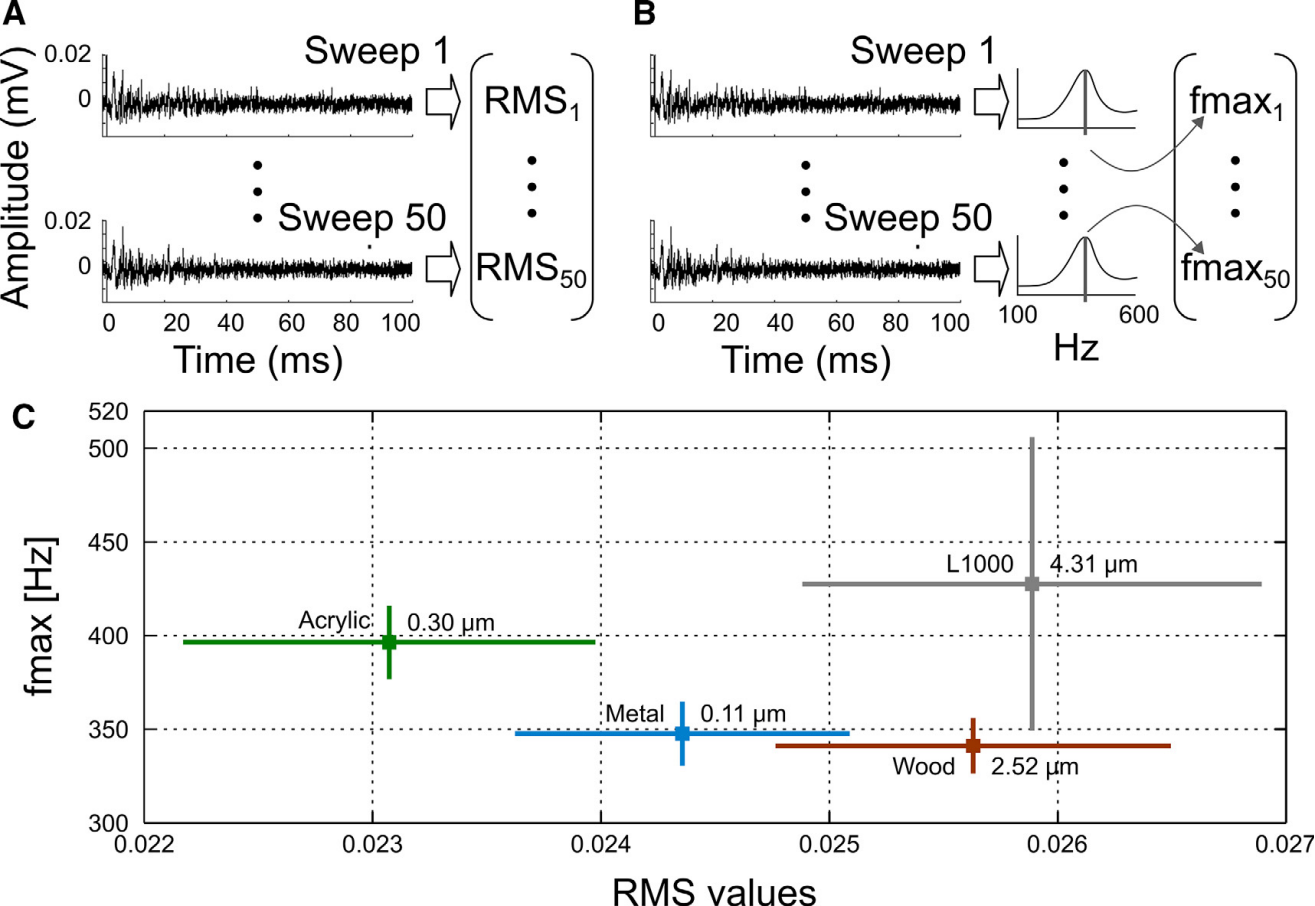
Amplitude and spectral information of afferent discharges at different sweep situations. (A) Determination of RMS values set belonging to a sweep situation. (B) Determination of fmax values set belonging to a sweep situation. (C) RMS values versus fmax values for each sweep situation.

RMS and fmax values were calculated from each afferent activity recording. Thus, each experimental sweep situation was represented by 50 RMS values and 50 fmax values (Fig. 2A and B). Then, averages and standard deviations were calculated and graphically represented in Figure 2C. Sweep situations on acrylic, metal and wood differ significantly from each other in their RMS values, while the sweep situation on L1000 can be differentiated from metal and wood in its fmax values. More importantly, experimental situations that are indistinguishable in their RMS values may be distinguishable in their fmax values, for example, L1000 versus wood situations.

**Figure 3:**
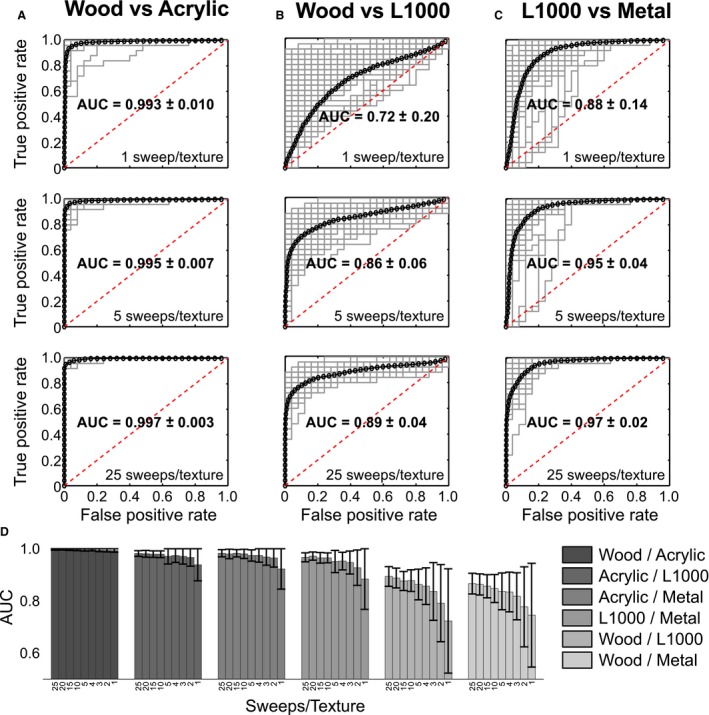
Analysis of ROC curves. (A) ROC curves obtained from classification processes of wood versus acrylic. Average ROC curves of 100 classification processes (training and validation) are represented by black circle‐lines [algorithm 4 of Fawcett (2006)], whereas some ROC curves are in gray lines (AUC >0.5). Three classification situations are shown: upper, using features of just a sweep/texture; middle: 5 sweeps/texture; and bottom: 25 sweeps/texture. (B) and (C) idem to A) for classification processes of wood versus L1000 and L1000 versus metal, respectively. (D) AUC values for all pairwise comparisons versus the amount of sweeps used in the training phase.

Figure 3 shows ROC curves of three classification processes: wood versus acrylic, wood versus L1000, and L1000 versus metal. Average ROC curves of 100 classification processes (training and validation) are represented by black circle‐lines, whereas some ROC curves are in gray lines (AUC > 0.5). The amount of features observed by the SP were those belonging to 1, 5, and 25 sweeps per texture.

High classification performances are observed for wood versus acrylic sweep situations (Fig. 3A). For this case, the SP achieves a high classification performance (AUC = 0.993 ± 0.010) just by observing one feature per experimental situation (Fig. 3A – upper). A greater quantity of observations does not improve the performance of the SP (Fig. 3A – middle and bottom). These results suggest that neuronal responses exhibit a high degree of specificity that allows a higher level of discrimination (measured by AUC) when both stimuli (sweeps on wood and acrylic) are presented to the vibrissal system.

Wood versus L1000 were the experimental situations with the lowest classification performance. The SP achieves a classification performance (AUC = 0.72 ± 0.20) just by observing one feature per experimental situation (Fig. 3B – upper). The average AUC value was greater than 0.5 (random classification) despite the poor performance. A significant increase in the average AUC value is observed from five sweeps per texture in the training phase (Fig. 3B – middle). Then, a greater quantity of observations does not improve the performance of the SP (Fig. 3B –bottom). Similar results are observed for L1000 versus metal (but with higher AUC values) (Fig. 3C).

Figure 3D shows average AUC values and their corresponding standard deviations for all pairwise comparisons. After 5 to 10 sweeps/texture, the discrimination results do not improve and remain stable.

These results suggest that the specificity of the peripheral vibrissal system, quantified through neuronal responses, easily permits the discrimination between perceived stimuli, and that it can be quantified through an ROC curve analysis (AUC values). Furthermore, it has also been found that such specificity makes a linear binary classifier capable of detecting differences between stimuli with five sweeps at most.

## Discussion

It is known that, behaviorally, rats keep their whiskers in contact with discriminanda for several hundreds of milliseconds, during which time the animals repetitively sweep their vibrissae across the surface at a dominant frequency of 8 Hz (Carvell and Simons 1990). These observations imply that the rat needs an average of 5 to 6 sweeps to achieve recognition of a texture.

In this study, we have analyzed the specificity of the peripheral vibrissal system through the classification performance of a binary classifier trained to identify the stimulus origin. Here, we show that the peripheral vibrissal system responds with high specificity, that is, few vibrissal sweeps are necessary to achieve a high classification performance. The specificity of the peripheral level makes the neural responses to be different enough to allow a simple linear classifier to discriminate between two surfaces with very similar roughness (e.g., sweeps on wood and L1000) with five vibrissal sweeps at most. These results are consistent with those observed by (Carvell and Simons 1990) at behavioral level. Thus, a close relationship between neural and behavioral observations is evidenced.

### Peripheral afferent responses

The afferent activity is composed of activities of approximately 200 myelinated axons, and it is the result of phase summation and cancellation of single fiber potentials with amplitudes that depend on morphological features of active fibers (Farfán et al. 2011). Our hypothesis is that the changes of afferent signal amplitude (RMS value) and frequency components are related to different levels of activation and synchronization of mechanoreceptors; being these last ones dependent on roughness of surfaces. Here, the RMS and fmax values were employed in the same way as they were used by Albarracín et al. (2006). Previous studies have demonstrated the existence of specific patterns into peripheral afferent activity which provide accurate information about the palpated texture (Farfán et al. 2013). Such temporal patterns could reveal functional characteristics of specificity higher than those observed here. However, determining the afferent signal features that represent more accurately the applied stimulus (such as RMS, fmax, time‐frequency component, electrophysiological events, and others) was not of concern in this study. It is important to note that the RMS and fmax are two features that give an overview of the signal, that is, they are an average of the stimulus obtained. Despite this, these features convey sufficient information to classify between stimuli. This suggests that the textural stimulation set (widely used in the field) falls short of challenging the perceptual capabilities of the vibrissal system.

## Conclusion

In this study, we analyzed the functional neuronal responses specificity of the peripheral vibrissal system. We characterized this by the area under the ROC curve obtained from the performance of a classifier between two different stimulus origins. Functional characteristics, such as acuity measure of texture, encoding and information measures of neural encoding were proposed previously, but no study, had provided a measure of the amount of sweeps required to determine the origin of other stimuli.

## Conflict of Interest

None declared.
